# Clinical evolution and mortality of critically ill patients with SARS-CoV-2 pneumonia treated with remdesivir in an adult intensive care unit of Paraguay

**DOI:** 10.1186/s12879-023-08917-2

**Published:** 2024-01-02

**Authors:** Jessica Figueredo, Lorena Fontclara Lopez, Belinda Figueredo Leguizamon, Margarita Samudio, Marcelo Pederzani, Federico Fretes Apelt, Patricia Añazco, Ricardo Caballero, Hugo Bianco

**Affiliations:** 1 Adult Intensive Care Department, Facultad de Ciencias Médicas, Hospital de Clínicas, Universidad Nacional de Asunción, San Lorenzo, Paraguay; 2 Critical Medicine and Intensive Care, Facultad de Ciencias Médicas, Universidad Nacional de Asunción, San Lorenzo, Paraguay

**Keywords:** Remdesivir, Mortality, COVID-19, Intensive care, ARDS

## Abstract

**Background:**

The health crisis due to Covid-19 led to the search for therapeutics that could improve the evolution of the disease. Remdesivir, an antiviral that interferes with viral replication, was one of the first to be used for the treatment of this pathology.

**Objective:**

To determine clinical course and mortality of patients with severe SARS-CoV‐2 pneumonia treated with remdesivir, in comparison of those who didn’t receive the medication.

**Patients and methods:**

Retrospective cohort study, with medical records review of COVID-19 patients, between August 2020 and August 2021. The subjects were divided into two groups, those who received remdesivir before or after admission to intensive care and those who didn’t. The primary outcome variable was mortality in intensive care.

**Results:**

Of 214 subjects included, 109 (50,9%) received remdesivir. The median of days for the drug administration was 8 (2-20), IQR: 3. The bivariate analysis prove that the use of remdesivir was related with lower risk of develop Acute Respiratory Distress Syndrome (ARDS) (p = 0,019; OR: 0,521) and lower requirement of mechanical ventilation (p = 0,006; OR:0,450). Additionally, patients treated with remdesivir develop less kidney injury (p = 0,009; OR: 0,441). There was a total of 82 deaths, 29 (26,6%) in the remdesivir group and 53 (50,5%) in the control group [p < 0,001; OR: 0,356 (0,201-0,630)]. All the risk factors associated with mortality in the bivariate analysis were entered into the multivariate analysis by logistic regression, the use of remdesivir remained associated as an independent protective factor to mortality (p = 0.034; OR: 0.429).

**Conclusion:**

Critically ill patients with SARS-CoV-2 pneumonia treated with remdesivir had a lower risk of death and need for mechanical ventilation and develop less ARDS as compared to the control group. No differences were found in the presentation of adverse effects.

## Introduction

COVID-19 was first identified as an emergent infectious disease in December 2019, subsequently distributed all over the world. Pandemic declared in March 2020 leads to a profound and negative impact in health care system an economic in general [1]. The health crisis and the loss of thousands of lives forced the search for therapies that may improve the evolution of the disease, with antiviral such as remdesivir being the first to be used [2].

Remdesivir is a nucleotide prodrug, intracellularly metabolized to active triphosphate that inhibits RNA polymerase, interfering with viral replication [3]. This activity led to its use in patients with SARS-CoV-2 infection, in the absence of other effective treatments, being approved for urgent use in May 2020 [4, 5]. However, remdesivir pharmacodynamic and pharmacokinetic inside respiratory system and other infected organs of critical ill patients with COVID-19 remains largely unknow [4]. A significant number of studies have been conducted to test the clinical benefits and safety of this drug, leading to controversial results [6, 7]. Some studies mention its effectiveness in shortening the disease and reducing mortality (especially when administered in the first days of illness), [1, 8, 9] while others have not found a significant change in evolution or outcome when using it, mentioning the appearance of adverse effects [10, 11].

Few studies have been reported in Paraguay regarding critically ill patients with SARS-CoV-2 pneumonia [12], this study was conducted during the first and second waves of COVID-19 in Paraguay [13], with the predominant circulation of the gamma variant [14], this study aimed to determine the clinical course and mortality of severe pneumonia SARS-CoV-2 patients treated with remdesivir, in comparison with those who did not receive this medication.

## Materials and methods

### Study area

Adult Intensive Care Unit of the Hospital de Clínicas, depending of the Facultad de Ciencias Médicas - Universidad Nacional de Asunción (Paraguay), a tertiary referral public hospital.

### Design and study population

Retrospective cohort study with medical records review of all the COVID-19 patients, admitted to ICU over 18 years of age, confirmed by RT-PCR, in nasopharyngeal swab samples obtained between August 2020 and August 2021 until ICU discharge. Incomplete medical records and patients with less than 48 h of hospitalization, as well as readmissions, were excluded.

Subjects were distributed into two groups, those who received remdesivir before or after ICU admission, with an initial intravenous dose of 200 mg (at the first day), following 100 mg/day for four days (total five days) and those who did not received the drug because they were admitted to the ICU, two weeks after the onset of symptoms and/or had financial difficulties in acquiring the medication, at the beginning of the pandemic the institution did not provide the medication, and only did it in the last two months of the study).

### Data collection

Data were obtained from medical records and register in Google form®. The main outcome variable was ICU mortality. In addition, sociodemographic variables, morbidity, oxygenation status at admission expressed by SpO_2_/FIO_2_ ratio (pulse oximetric saturation/fractional inspired oxygen) in non-ventilated patients, and PaO_2_/FIO_2_ ratio (arterial oxygen partial pressure/fractional inspired oxygen) in intubated patients with mechanical ventilation (MV) were studied. SOFA initial (Sequential Organ Failure Assessment at admission), APACHE II score (*Acute Physiology And Chronic Health Evaluation*) at admission were classified in APACHE II > 20 and APACHE II ≤ 20. Variables as lengths days of stay, pharmacologic treatment (corticosteroid therapy, anticoagulation, convalescent plasma, tocilizumab), ARDS, MV, NIMV (noninvasive mechanical ventilation), days of MV, use of vasoactive drugs, prone position, intercurrent infections, kidney and hepatic injury, hemodialysis requirement), were also register.

### Statistical analysis

The data recorded in the Google form® were downloaded into Microsoft Excel (2017) format and analyzed using SPSS Inc. v. 12.5 (Chicago, Ill., USA). To analyze the baseline characteristics and outcomes between the two groups, the Chi-square test was used to compare qualitative variables, and the t-test or Mann-Whitney test, as appropriate, was used to compare means or medians, at a significance level of 0.05. All risk factors associated with mortality in the univariate analysis were entered into the multivariate analysis using logistic regression.

## Results

A total of 214 subjects were included in the study, of them 109 (50.9%) received remdesivir. The median illness time at which the study drug was administered was 8 days (range: 2–20), with an IQR of 3.

The comparison of clinical characteristics, treatment, and evolution between both groups is presented in Table 1. There was a significant difference between the groups in terms of mean age and median severity scores at admission.

**Table 1:** Clinical characteristics, and treatment of patients with COVID-19 in the Adult Intensive Care Department of the Hospital de Clínicas during the first and the second waves of COVID-19 between August 2020 and August 2021. San Lorenzo. Paraguay (n=214)

Variable	Total n = 214	Remdesivir n = 109	No Remdesivir n = 105	OR (CI 95%)	*P*
Male sex, n (%)	124 (57,9)	58 (53,2)	66 (62,9)	0.672 (0.389-1.160)	0,153
Age (years), mean ± SD	53,10±13,11	52,23±13,1	54,2±13,0		0,001
Vaccinated patients	8 (3,7)	3 (2,7)	5 (4,7)	0.566 (0.131-2.430)	0.340
Comorbidities					
Hypertension, n (%)	107 (50)	55 (50,5)	52 (49,5)	10.3 (0.607-1.774)	0,891
Obesity, n (%)	102 (47,7)	60 (55,0)	42 (40,0)	1.837 (1.067-3.161)	0,028
Diabetes mellitus, n (%)	56 (26,2)	24 (22,0)	32 (30,5)	0.644 (0.348-1.191)	0,159
Chronic renal failure, n (%)	12 (5,6)	5 (4,6)	7 (6,7)	0.673 (0.207-2.191)	0,509
Cardiac disease, n (%)	12 (5,6)	7 (6,4)	5 (4,8)	1.372 (0.422-4.468)	0,598
Severity Score					
APACHE II, median (IQR)	12 (8)	10 (6)	13 (9)		<0.001
SOFA initial, median (IQR)	4 (5)	3 (3)	4 (5)		<0.001
Treatment					
Corticosteroid therapy	183 (85,5)	93 (85,3)	90 (85,7)	0.969 (0.452-2.075)	0,935
Anticoagulation					
Prophylaxis	42 (19,6)	22 (20,1)	20 (19)	1.075 (0.547-2.11)	0.834
Therapeutic	154 (72,0)	77 (70,6)	77 (70,6)	0.875 (0.481-1.590)	0,661
Convalescent plasma	48 (22,4)	31 (28,4)	17 (16,2)	2.05 (1.058-4.002)	0,032
Tocilizumab	20 (9,3)	9 (8,3)	11 (10,5)	0.769 (0.305-1.939)	0,577

OR. Odds ratio, CI: confidence interval, SD: standard deviation, IQR: interquartile range. APACHE II: *Acute Physiology and Chronic Health Evaluation*, SOFA initial: *Sequential Organ Failure Assessment*, SpO_2_/FIO_2_: Pulse oximetric saturation/ Fractional inspired oxygen_,_ PaO_2_ /FIO_2_ :Arterial oxygen partial pressure/ Fractional inspired oxygen, MV: Mechanical ventilation, ARDS: Acute Respiratory Distress Syndrome

In the bivariate analysis, the use of remdesivir was associated with a lower risk of developing ARDS (p = 0.019; OR: 0.521) and the need for mechanical ventilatory support (p = 0.006; OR: 0.450). Additionally, individuals treated with remdesivir had a lower risk of developing kidney injury (p = 0.009; OR: 0.441), without implying an increased need for hemodialysis sessions in the non-remdesivir group (p = 0.382) (Table 2).

There was a total of 82 deaths, 29 (26.6%) in the remdesivir group, and 53 (50.5%) in the group that did not receive the antiviral, with a significant difference [*p*-value < 0.001; OR: 0.356 (0.201–0.630)].

**Table 2:** Evolution of patients with COVID-19 in the Adult Intensive Care Units of the Hospital de Clínicas between August 2020 and August 2021. San Lorenzo. Paraguay (n = 214)

Variable	Remdesivir n = 109	No Remdesivir n = 105	OR (CI 95%)	*P*
Length of stay in the ICU, median (IQR)	11 (11)	10 (10)	-	0.150
SaO_2_/FIO_2,_ median (IQR)	160 (63)	146 (73)	-	0.353
PaO_2_ /FIO_2,_ median (IQR)	102 (77)	100 (63)	-	0.671
MV at admission, n (%)	40 (36,7)	61 (58,1)	0.4182 (0.2414–0.7244)	0.001
NIMV at admission, n (%)	1 (0,9)	4 (3,8)	0.2338 (0.026–2.1271)	0.1730
MV total, n (%)	59 (54.1)	76 (72.4)	0.450 (0,255-0,796)	0.006
Days of MV, median (IQR)	13.5 (13)	11 (11)	-	0.039
ARDS, n (%)	43 (43.9)	68 (60.0)	0.521 (0.302-0.900)	0.019
Prone position, n (%)	56 (57.7)	71 (61.2)	0.866 (0.500-1.499)	0.607
Norepinephrine, n (%)	51 (52.0)	73 (62.9)	0.639 (0.370–1.104)	0.108
Hemodialysis, n (%)	13 (13.3)	11 (9.5)	0.460 (0.623–3.424)	0.382
Kidney injury	23/93 (24.7)	41/96 (42.7)	0.441 (0.237–0.820)	0.009
Hepatic injury	9/47 (19.1)	5/57 (8.8)	2.463 (0.764–7.940)	0.123
Intercurrent infection, n (%)	70 (71.4)	95 (81.9)	0.553 (0.290–1.053)	0.069
Death, n (%)	29 (26.6)	53 (50.5)	0.356 (0.201–0.630)	< 0.001

OR. Odds ratio, CI: confidence interval, IQR: interquartile range, SpO_2_/FIO_2_: Pulse oximetric saturation/ Fractional inspired oxygen, PaO_2_ /FIO_2_: Arterial oxygen partial pressure/ Fractional inspired oxygen, MV: Mechanical ventilation, NIMV; noninvasive mechanical ventilation, ARDS: Acute Respiratory Distress Syndrome

A bivariate analysis of the different factors associated with mortality was also performed, with remdesivir included as one of the variables (Table 3).

**Table 3:** Bivariate analysis of clinical characteristics, treatment, and evolution of patients with COVID-19 in the Adult Intensive Care Unit of the Hospital de Clínicas between August 2020 and August 2021. San Lorenzo. Paraguay (n = 214)

Variable	Deceased (n = 82)	Survivors (n = 132)	*p*-value
Age > 60	40 (48.8)	39 (29.5)	0.005
Male sex	51 (62.2)	73 (55.3)	0.321
Obesity	40 (48.8)	62 (47.0)	0.797
Diabetes mellitus	29 (35.4)	27 (20.5)	0.016
Cardiac disease	8 (9.8)	4 (3.0)	0.076
Hypertension	46 (56.1)	61 (46.2)	0.160
Chronic renal failure	9 (11)	3 (2.3)	0.007
APACHE II > 20	22 (26.8)	6 (4.5)	< 0.001
Renal injury	31/68 (45.6)	33/121 (27.3)	0.011
Hepatic injury	9/39 (23.1)	5/65 (7.7)	0.026
Remdesivir	29 (35.4)	80 (60.6)	< 0.001
Received remdesivir until the 8th day of illness	13 (26.0)	42 (32.1)	< 0.001
Received remdesivir after the 8th day of illness	15 (18.5)	37 (28.2)	
No remdesivir	53 (65.4)	52 (39.7)	
Plasma	18 (22.0)	30 (22.7)	0.895
Tocilizumab	5 (6.1)	15 (11.4)	0.198
MV	81 (98.8)	54 (40.9)	< 0.001
ARDS	46 (56.8)	66 (50.0)	0.335
Norepinephrine	80 (97.6)	44 (33.3)	< 0.001
Hemodialysis	22 (26.8)	2 (1.5)	< 0.001
Intercurrent infections	69 (84.1)	96 (72.7)	0.053

*APACHE II: Acute Physiology and Chronic Health Evaluation, MV: Mechanical ventilation, ARDS: Acute Respiratory Distress Syndrome*

All risk factors associated with mortality in the bivariate analysis, were included in the multivariate analysis through logistic regression.

In the multivariate analysis, the remdesivir treatment was independently associated with lower mortality risk (p = 0.034; OR 0.429). (Table 4)

**Table 4:** Multivariate analysis. Evolution of patients with COVID-19 in the Adult Intensive Care Unit of the Hospital de Clínicas between August 2020 and August 2021. San Lorenzo. Paraguay (n = 214)

Variables	*p*-value	OR (CI 95%)		
Age > 60	0.001	4.401	1.792	10.811
APACHE > 20	0.024	3.868	1.194	12.530
Norepinephrine	< 0.001	92.418	20.053	425.915
Remdesivir	0.034	0.429	0.196	0.938
Diabetes mellitus	0.747	1.214	0.334	4.617
Renal injury	0.327	1.788	0.559	5.717
Hepatic injury	0.346	2.643	0.349	19.988

*OR: Odds ratio* CI: confidence interval, *APACHE II: Acute Physiology and Chronic Health Evaluation*,

## Discussion

In this study, the evolution and mortality of adult patients with severe SARS-CoV-2 pneumonia admitted to intensive care and treated with remdesivir were evaluated at a reference hospital in Paraguay from August 2020 to August 2021. Patients who received the antiviral showed a lower risk of death and developing ARDS, as well as reduced need for mechanical ventilatory support compared to the control group. Patients treated with remdesivir also had a lower risk of developing kidney injury.

Our study includes critically ill patients with bilateral SARS-CoV-2 pneumonia, all of them required oxygen therapy. We found a significant difference in mortality rate between the patients who received remdesivir and the control group (26,6% vs. 50,5%). Numerous studies have reported results on the use of this medication [11, 13–15], but concerning critically ill patients are scarce. Metha et al. [16] mention a 22% mortality rate in the overall remdesivir group and a 30,8% fatal outcome in the subgroup of critically ill patients, reporting a mortality benefit with the use of this antiviral and emphasizing the importance of administering it as early as possible to achieve better outcomes. Zerbit et al. [17] also report a benefit with the use of remdesivir in critically ill patients with a Sequential Organ Failure Assessment Score (SOFA) less than 10, but remarkably, the mortality in this group is only 14%.

There are several possible explanations for the decrease in mortality in the remdesivir group of our critically ill patients, firstly, the mean age is 53 years, this study population is relatively younger than critically ill COVID-19 patients reported in other studies [14, 18, 19]. Another reason could be that the median number of days of illness (symptom onset) at which the drug was administered was 8 days. A high viral load of SARS-CoV-2, massive replication with persistent high viremia, is associated with the severity of the condition, hyperinflammation with damage to multiple organs, and increased mortality [20–22]. The clinical benefits observed with remdesivir are attributed to the inhibition of viral replication, leading to a reduction in viral load and improvement of lung lesions. Therefore, the early initiation of remdesivir has contributed to the positive outcomes obtained, as mentioned in some studies [16, 23], compared to other works that may have administered the drug later to achieve a significant change in the course of the disease [11, 14]. Finally, in this study, a high percentage of patients in both groups have received systemic corticosteroids, one of the therapeutic measures that has irrefutably demonstrated its utility in this pathology [24]. A Danish study has shown a significant reduction in 30-day mortality with the combined use of remdesivir and dexamethasone in mechanically ventilated patients [25], as well as the work reported by Pilgram et al. [26], which also mentions a decrease in mortality with this combination.

This could explain why early studies, lacking the concomitant administration of steroids as part of their protocols, like the Beigel et al. study [11] with steroids administered in only 22% of patients, Wang et al. [14] with 38%, and the World Health Organization’s Solidarity Trial [10] with 48%, may have been adversely affected in terms of their results on mortality and progression to invasive mechanical ventilation.

In this study, a very small percentage (less than 10%) of patients corresponding to both groups (those treated and not treated with remdesivir) received Tocilizumab, so it would not constitute a variable that affects the evolution of the patients, with respect of the use of convalescent plasma, its efficacy has been shown to be low in critically ill COVID-19 patients [27, 28] therefore we do not consider that both therapeutics constitute confusion variables.

The use of remdesivir was also associated with a lower risk of developing ARDS compared to patients who did not receive this antiviral in this study. Additionally, a higher need for MV was observed in the control group. It has been pointed out that administering remdesivir within an optimal therapeutic window, meaning in the early stages of the disease, with moderate symptoms and before the activation of the inflammatory cascade, may provide greater benefits by preventing the progression of lung injuries and the need for oxygen therapy through high-flow nasal cannula or MV [29]. One study mentioned that treatment with remdesivir was associated with a lower likelihood of requiring high-flow oxygen supplementation and invasive MV when compared to standard treatment [30].

This was supported by a systematic review and meta-analysis, which revealed that the odds of mechanical ventilation were significantly lower in the remdesivir group than in the control group [9]. Similarly, Alexander et al. [23] reported that the progression to non-invasive ventilation was lower in the remdesivir group; however, in this study, the progression from non-invasive to invasive ventilation was higher in the remdesivir group, likely indicating that once lung damage is established in the late stage of hyperinflammation, remdesivir may no longer be able to reverse the pulmonary injuries.

In this study, no significant adverse effects were detected in the remdesivir group. Hepatic injury occurred in both groups without significant differences, while kidney injury was more noticeable in the control group, as also reported by the team of Zerbit et al. [17]. In fact, there are studies that have reported that SARS-CoV-2 itself is associated with hepatic and kidney injuries, which could explain the observations in the control group [31–34]. Regarding the safety of remdesivir, several studies have reported no significant differences in the occurrence of adverse effects between the study groups (remdesivir and control) [11, 13, 15, 33, 35].

This study has limitations such as the retrospective design, which has made it impossible to monitor some variables. Additionally, it was conducted in a single hospital center, which has limited the sample size. Another limitation was that at the beginning of the study some patients were indicated Remdesivir administration, yet were unable to receive it due to financial difficulties in acquiring it. The institution provided the drug only during the final two months of the study. Despite these limitations, significant differences in favor of remdesivir have been observed. It is important to mention that the challenges posed by the pandemic, such as limited economic resources for optimizing therapies as well as those of human resources, may have affected patient care. These factors undoubtedly influenced the outcomes, but they have not been aborded in this study.

## Conclusion

This group of critically ill patients with SARS-CoV-2 pneumonia were mostly men with comorbidities in the fifth decade of life, treated with remdesivir, presented a lower risk of mortality compared to those who were not medicated with the antiviral. This result is similar to reports that have reported the benefits observed with the administration of this medication. Additionally, this group of patients developed less ARDS and had a lower need for MV compared to the control group. No significant adverse effects were found from this medication, with the most notable impact being on kidney involvement caused by SARS-CoV-2.

## Data Availability

The datasets generated and/or analyzed during the current study are not publicly available, but are available from the corresponding author on reasonable request.

## References

[1] Angamo MT, Mohammed MA, Peterson GM. Efficacy and safety of remdesivir in hospitalised COVID-19 patients: a systematic review and meta-analysis. Infection. 2022. 10.1007/s15010-021-01671-0 PMC8325414 34331674

[2] Wu Z, Han Z, Liu B, Shen N (2022). Remdesivir in treating hospitalized patients with COVID-19: a renewed review of clinical trials. Front Pharmacol.

[3] Saber-Ayad MA-O, Saleh MA-O, Abu-Gharbieh EA-O (2020). The Rationale for potential pharmacotherapy of COVID-19. Pharmaceuticals (Basel).

[4] National Institute of Health. COVID-19 Treatment Guidelines. 2021. https://www.covid19treatmentguidelines.nih.gov/whatsnew/. Consulted 25 April 2023.

[5] Rubin D, Chan-Tack K, Farley J, Sherwat A (2020). FDA approval of remdesivir - a step in the right direction. N Engl J Med.

[6] Okoli GN, Rabbani R, Copstein L, Al-Juboori A, Askin N, AbouSetta AM (2021). Remdesivir for coronavirus Disease 2019 (COVID-19): a systematic review with meta-analysis and trial sequential analysis of randomized controlled trials. Infect Dis.

[7] Shrestha DB, Budhathoki P, Syed NI, Rawal E, Raut S, Khadka S (2021). Remdesivir: a potential game-changer or just a myth? A systematic review and meta-analysis. Life Sci.

[8] Al-Abdouh A, Bizanti A, Barbarawi M, Jabri A, Kumar A, Fashanu OE (2021). Remdesivir for the treatment of COVID-19: a systematic review and meta-analysis of randomized controlled trials. ContempClin Trials.

[9] Reddy Vegivinti CT, Pederson JM, Saravu K, Gupta N, Barrett A, Davis AR (2012). Remdesivir therapy in patients with COVID-19: a systematic review and meta-analysis of randomized controlled trials. Ann Med Surg.

[10] Pan H, Peto R, Henao-Restrepo A-M, WHO Solidarity Trial Consortium (2021). Repurposed antiviral Drugs for COVID-19: interim WHO solidarity trial results. N Engl J Med.

[11] Beigel JH, Tomashek KM, Dodd LE, Mehta AK, Zingman BS, Kalil AC (2020). Remdesivir for the treatment of covid-19 - final report. N Engl J Med.

[12] Figueredo B, Samudio M, Fretes F, Delgado R, Ibarra D, Pederzani M, Fontclara L, Caballero R, Bianco H (2023). Factores asociados a mortalidad en pacientes críticos con Covid-19 en un centro universitario de Paraguay. Revista Chil De Infectología.

[13] MSPyBS, Reporte Paraguay. MSPBS COVID19 [Internet]. https://www.mspbs.gov.py. 2022 [citado el 9 de noviembre de 2023]. Disponible en: https://www.mspbs.gov.py/reportes-covid19.html.

[14] Martinez M, Nguyen P-V, Su M, Cardozo F, Valenzuela A, Franco L (2022). SARS-CoV-2 variants in Paraguay: detection and surveillance with an economical and scalable molecular protocol. Viruses [Internet].

[15] Alibrahim RS, Elmekaty EZ, Elmekaty MZI, Edbais M, Alkhatib M, Daghfal J, Almaslamani MA, Omrani AS (2022). Remdesivir for patients with coronavirus Disease 2019 Pneumonia requiring high oxygen support. Qatar Med J.

[16] Wang L-Y (2020). Remdesivir and COVID-19. Lancet.

[17] Amstutz A, Speich B, Mentré F, et al. Effects of Remdesivir in Hospitalized patients with COVID-19: systematic review and Individual Patient Data Meta-Analysis of Randomized clinical trials. SSRN; 2022. 10.2139/ssrn.4244759.

[18] Mehta RM, Bansal S, Bysani S, Kalpakam H (2021). A shorter symptom onset to remdesivir treatment (SORT) interval is associated with a lower mortality in moderate-to-severe COVID-19: a real-world analysis. Int J Infect Dis.

[19] Zerbit J, Detroit M, Chevret S, Pene F, Luyt CE, Ghosn J, Eyvrard F, Martin-Blondel G, Sarton B, Clere-Jehl R, Moine P, Cransac A, Andreu P, Labruyère M, Albertini L, Huon JF, Roge P, Bernard L, Farines-Raffoul M, Villiet M, Venet A, Dumont LM, Kaiser JD, Chapuis C, Goehringer F, Barbier F, Desjardins S, Benzidi Y, Abbas N, Guerin C, Batista R, Llitjos JF, Kroemer M (2022). Remdesivir for patients hospitalized with COVID-19 severe Pneumonia: a National Cohort Study (Remdeco-19). J Clin Med.

[20] Grasselli G, Zangrillo A, Zanella A, Antonelli M, Cabrini L, Castelli A (2020). Baseline characteristics and outcomes of 1591 patients infected with SARS-CoV-2 admitted to ICUs of the Lombardy region, Italy. JAMA.

[21] Cummings MJ, Baldwin MR, Abrams D, Jacobson SD, Meyer BJ, Balough EM (2020). Epidemiology, clinical course, and outcomes of critically ill adults with COVID-19 in New York City: a prospective cohort study. Lancet.

[22] Fajnzylber J, Regan J, Coxen K, Corry H, Wong C, Rosenthal A (2020). SARS-CoV-2 viral load is associated with increased Disease severity and mortality. Nat Commun.

[23] Hagman K, Hedenstierna M, Rudling J, Gille-Johnson P, Hammas B, Grabbe M, Jakobsson J, Dillner J, Ursing J (2022). Duration of SARS-CoV-2 viremia and its correlation tomortality and inflammatory parameters in patients hospitalized for COVID-19: a cohort study. Diagn Microbiol Infect Dis.

[24] Tan C, Li S, Liang Y, Chen M, Liu J (2020). SARS-CoV-2 viremia may predict rapid deterioration of COVID-19 patients. Braz J Infect Dis.

[25] Alexander H, Gunasekaran K, Sara John J, Gracelin Princy Zacchaeus N, Samuel P, Jasmine S, Christopher DJ, Pichamuthu K, Rupali P. Evaluation of Remdesivir to the outcomes of hospitalized patients with COVID-19 infection in a tertiary-care hospital in southern India. J Infect Dev Ctries. 2023;17(3):304–310. 10.3855/jidc.16642. PMID: 37023432. 10.3855/jidc.16642 37023432

[26] Sterne JAC, Murthy S, Slutsky AS, Villar J, The WHO Rapid Evidence Appraisal for COVID-19 Therapies Working Group (2020). Association between administration of systemic corticosteroids and mortality among critically ill patients with COVID-19: a meta-analysis. JAMA.

[27] Writing Committee for the REMAP-CAP, Investigators, Abdelhady H, Abdelrazik M, Abdi Z, Abdo D, Abdulle A et al. Effect of convalescent plasma on organ support–free days in critically ill patients with COVID-19: A randomized clinical trial. JAMA [Internet]. 2021;326(17):1690. 10.1001/jama.2021.18178. 10.1001/jama.2021.18178 PMC8491132 34606578

[28] Simonovich VA, Burgos Pratx LD, Scibona P, Beruto MV, Vallone MG, Vázquez C et al. A randomized trial of convalescent plasma in Covid-19 severe pneumonia. N Engl J Med [Internet]. 2021;384(7):619–29. 10.1056/NEJMoa2031304. 10.1056/NEJMoa2031304 PMC7722692 33232588

[29] Benfield T, Bodilsen J, Brieghel C, Harboe ZB, Helleberg M, Holm C (2021). Improved survival among hospitalized patients with COVID-19 treated with remdesivir and dexamethasone. A nationwide population-based cohort study. Clin Infect Dis.

[30] Pilgram L, Appel KS, Ruethrich MM, Koll CEM, Vehreschild MJGT, de Miranda SMN, Hower M, Hellwig K, Hanses F, Wille K, Haselberger M, Spinner CD, Vom Dahl J, Hertenstein B, Westhoff T, Vehreschild JJ, Jensen BO, Stecher M. Use and effectiveness of remdesivir for the treatment of patients with covid-19 using data from the lean European Open Survey on SARS-CoV-2 infected patients (LEOSS): a multicentre cohort study. Infect 2023 Feb 10:1–17. 10.1007/s15010-023-01994-0. Epub ahead of print. PMID: 36763285; PMCID: PMC9913009. 10.1007/s15010-023-01994-0 PMC9913009 36763285

[31] Gottlieb RL, Vaca CE, Paredes R, Mera J, Webb BJ, Perez G, Oguchi G, Ryan P (2022). GS-US-540-9012 (PINETREE) investigators. Early Remdesivir to prevent progression to severe Covid-19 in outpatients. N Engl J Med.

[32] Angamo MT, Mohammed MA, Peterson GM (2022). Efficacy and safety of remdesivir in hospitalised COVID-19 patients: a systematic review and meta-analysis. Infection.

[33] Ronco C, Reis T, Husain-Syed F (2020). Management of Acute kidney Injury in patients with COVID-19. Lancet Respir Med.

[34] Zhang C, Shi L, Wang F-S (2020). Liver Injury in COVID-19: Management and challenges. Lancet Gastroenterol Hepatol.

[35] Ali K, Azher T, Baqi M, Binnie A, Borgia S, Carrier FM, Cavayas YA, Chagnon N, Cheng MP, the Canadian Critical Care Trials Group. Canadian Treatments for COVID-19 (CATCO); Association of Medical Microbiology and Infectious Disease Canada (AMMI) Clinical Research Network and. Remdesivir for the treatment of patients in hospital with COVID-19 in Canada: a randomized controlled trial. CMAJ. 2022;194(7):E242-E251. 10.1503/cmaj.211698. Epub 2022 Jan 19. PMID: 35045989; PMCID: PMC8863204. 10.1503/cmaj.211698 PMC8863204 35045989

